# The link between systemic inflammation and mental disorders: a study on CLR, depression, and anxiety in a US cohort

**DOI:** 10.3389/fpsyt.2025.1607982

**Published:** 2025-08-18

**Authors:** Yue Su, Yuhan Ge, Hui Yang, Guojie Zhai, Xiaolan Cheng

**Affiliations:** ^1^ School of Integrated Chinese and Western Medicine, Nanjing University of Chinese Medicine, Nanjing, China; ^2^ School of Health Preservation and Rehabilitation, Nanjing University of Chinese Medicine, Nanjing, China; ^3^ Department of Neurology, Suzhou Ninth People’s Hospital, Suzhou Ninth Hospital Affiliated to Soochow University, Suzhou, China

**Keywords:** C-reactive protein to lymphocyte ratio, depression, anxiety, inflammation, mental health, NHANES

## Abstract

**Background:**

Depression and anxiety are significant global health concerns, with systemic inflammation playing a critical role in their pathophysiology. Recent studies have highlighted the C-reactive protein to lymphocyte ratio (CLR) as a potential biomarker of inflammation that may be associated with these mental health conditions. However, the relationship between CLR and depression and anxiety, especially within a diverse population, remains underexplored.

**Methods:**

This study utilized data from the National Health and Nutrition Examination Survey (NHANES) (2015–2023) to examine the association between CLR and the prevalence of depression and anxiety. A total of 22,308 participants were included for depression analysis, and 16,138 participants were included for anxiety analysis. Depression was assessed using the PHQ-9, and anxiety was assessed through self-reported anxiety symptoms and medication use. CLR was calculated as the ratio of C-reactive protein to lymphocyte count, and logistic regression models were applied to analyze associations, adjusting for demographic and health-related variables.

**Results:**

Higher CLR levels were significantly associated with increased odds of depression (OR: 1.49; 95% CI: 1.25–1.78) and anxiety (OR: 1.13; 95% CI: 1.02–1.26) after full adjustment for confounders. Non-linear relationships were observed, with specific inflection points for both depression (CLR = 0.96) and anxiety (CLR = 0.88), beyond which the risk of mental health disorders increased sharply. Subgroup analyses revealed that younger individuals and those without hypertension showed stronger associations between CLR and depression.

**Conclusion:**

Elevated CLR is associated with an increased risk of depression and anxiety, suggesting the potential role of systemic inflammation in influencing mental health outcomes. CLR may serve as a useful biomarker for identifying populations at higher risk, underscoring the need for further research into early intervention strategies and targeted approaches to address systemic inflammation in mental health care.

## 1 Introduction

Depression and anxiety are among the most common mental disorders worldwide, affecting over 300 million people annually and contributing significantly to global disability and disease burden (1). These conditions are multifactorial, influenced by genetic, environmental, and biological factors, among which inflammation has emerged as a critical pathway linking physiological dysregulation to mental health disorders (2). Numerous studies have demonstrated that inflammatory biomarkers, such as C-reactive protein (CRP), are elevated in individuals with depression and anxiety, underscoring the role of systemic inflammation in the pathophysiology of these conditions (3, 4).

CRP, an acute-phase reactant produced by the liver in response to inflammation, has been widely utilized as a marker of systemic inflammation (5). Lymphocytes, a subset of white blood cells, are key mediators of immune responses, and their count often decreases during systemic inflammatory states (6). The C-reactive protein to lymphocyte ratio (CLR) has recently emerged as a novel inflammatory marker, reflecting both the pro-inflammatory and immunosuppressive aspects of systemic inflammation (7, 8). CLR has been linked to various chronic diseases, including cardiovascular disease, cancer, and autoimmune disorders (9, 10). However, its role in mental health conditions, particularly depression and anxiety, remains insufficiently explored. Emerging evidence suggests a bidirectional relationship between systemic inflammation and mental health. Pro-inflammatory cytokines can cross the blood-brain barrier, altering neurotransmitter metabolism, neural plasticity, and the hypothalamic-pituitary-adrenal (HPA) axis, all of which are implicated in the development of depression and anxiety (11, 12). Conversely, chronic psychological stress and mental health disorders can exacerbate systemic inflammation, creating a vicious cycle that perpetuates both physical and mental health deterioration (13). A growing body of research has underscored the significant role of inflammatory markers, such as interleukins (e.g., IL-6), tumor necrosis factor-alpha (TNF-α), and trace elements including zinc and copper, in the pathophysiology of affective disorders (14, 15). Elevated concentrations of these biomarkers have been robustly linked to an increased risk of both depression and anxiety, suggesting that systemic inflammation plays a pivotal role in the dysregulation of mood and emotional homeostasis (16). These inflammatory mediators are thought to influence mood regulation through complex interactions with neurotransmitter systems, including serotonin, dopamine, and glutamate, thereby exacerbating or perpetuating affective symptoms (17). The dysregulation of these systems, in turn, may contribute to the onset and persistence of depressive and anxiety disorders, highlighting the intricate connection between immune responses and emotional regulation. Moreover, these markers may serve not only as indicators of the inflammatory burden but also as potential therapeutic targets for the management of mood disorders, warranting further investigation into their role in both the pathogenesis and treatment of these conditions.

Despite these advances, the interaction between demographic factors, comorbidities, and inflammatory markers such as CLR in depression and anxiety is not well understood. The National Health and Nutrition Examination Survey (NHANES) provides a valuable resource to investigate these associations in a diverse, representative sample of the U.S. population. Utilizing NHANES data from 2015 to 2023, this study aims to examine the relationship between CLR and the prevalence of depression and anxiety, while accounting for potential confounders such as age, sex, socioeconomic status, and lifestyle factors (18).Subgroup and interaction analyses aim to identify vulnerable populations where the association between CLR and mental health outcomes may be particularly pronounced, advancing our understanding of mental health heterogeneity and informing targeted prevention and intervention strategies (19).

This study represents the first comprehensive exploration of CLR as an inflammatory biomarker for depression and anxiety in a nationally representative cohort. By integrating robust statistical modeling with population-based data, we aim to shed light on the inflammatory underpinnings of mental health disorders and provide novel insights into their prevention and management.

## 2 Methods

### 2.1 Study design and population

This cross-sectional study was conducted using data from the National Health and Nutrition Examination Survey (NHANES) between 2015 and 2023. NHANES employs a complex, multistage probability sampling design to collect representative health and nutritional data from the U.S. population.

**Figure 1:**
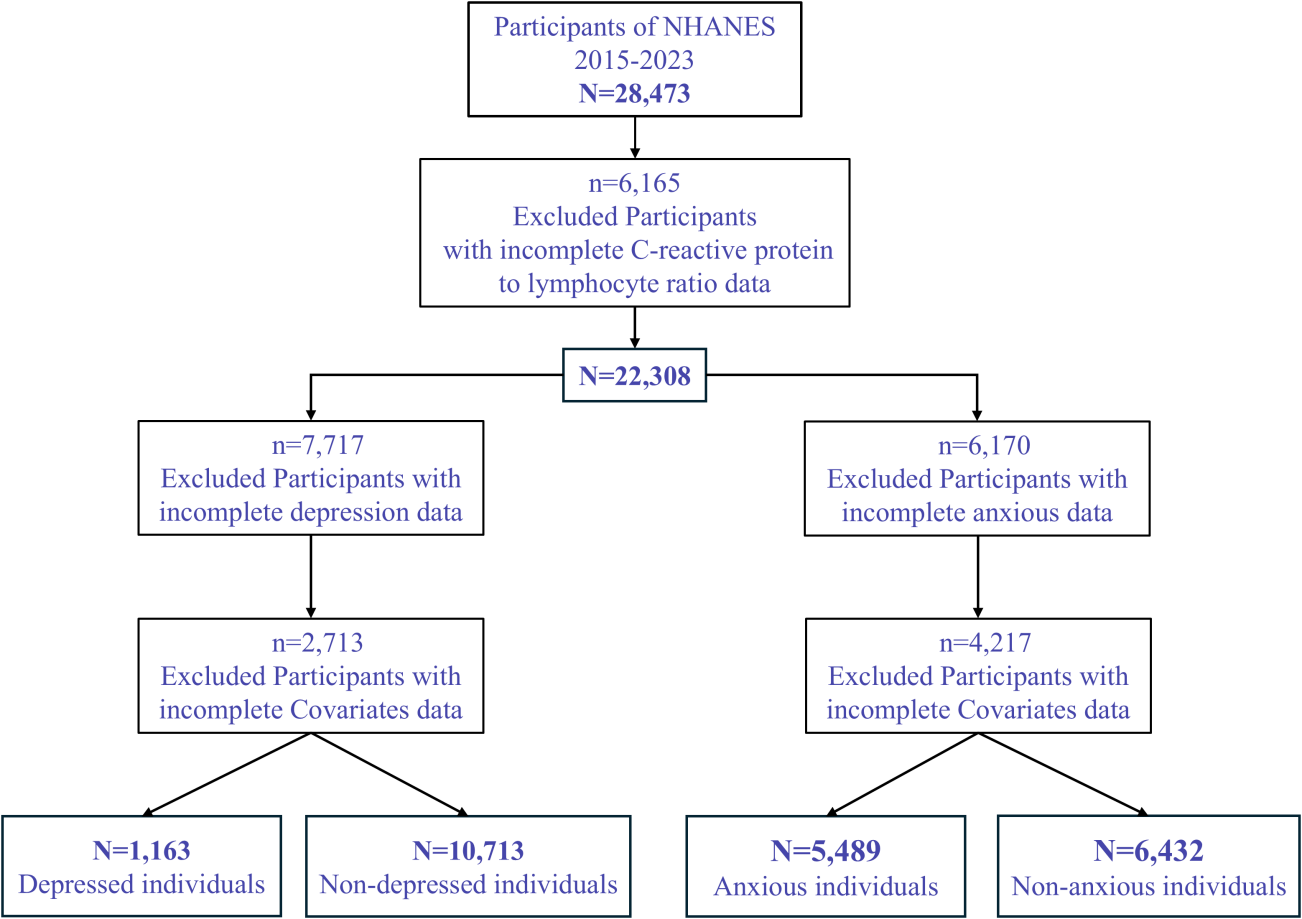
Flowchart of the screening process for the study population.

A total of 28,473 participants were initially included. After excluding 6,165 participants with missing data on C-reactive protein to lymphocyte ratio (CLR), 22,308 participants remained. For the depression analysis, 7,717 participants with missing depression data were further excluded, resulting in 14,591 individuals. An additional 2,713 participants with missing covariate data were excluded, yielding a final sample size of 11,878 individuals, including 1,163 participants with depression and 10,715 without (**Figure 1**).

For the anxiety analysis, 6,170 participants with missing CLR data were excluded, resulting in 16,138 participants. After excluding 4,217 participants with missing covariate data, the final analytic sample comprised 11,921 participants, including 5,489 individuals with anxiety and 6,430 without.

### 2.2 Definition of depression and anxiety

The 9-item PHQ-9 was used to assess depressive symptoms. PHQ-9 is well accepted as an accurate and reliable tool for screening depression. The total PHQ-9 score varies from 0 to 27, with higher scores indicating more severe depression. Compared with the clinical interview criteria, a PHQ score of ≥10 had a sensitivity of 85% and a specificity of 89% for major depressive disorder (MDD) (20). The PHQ-9 is a widely used tool for screening depression, but it is not a diagnostic tool and may not fully capture the complexity of depression. It is important to note that a score of ≥10 indicates potential major depressive disorder (MDD), but further clinical evaluation is required for a formal diagnosis. The limitations of self-reported data in assessing mental health symptoms should also be acknowledged.

Anxiety was assessed using self-reported frequency of anxiety-related feelings and anxiety medication use, as recorded in the NHANES mental health questionnaire. Participants were asked, “How often do you feel worried, nervous, or anxious? Would you say daily, weekly, monthly, a few times a year, or never?” Those who reported experiencing anxiety daily or weekly or who reported current use of anxiety medication were classified as having significant anxiety symptoms (21). This approach aligns with methodologies employed in prior studies and captures both self-reported emotional experiences and clinical management of anxiety.

### 2.3 Calculation of C-reactive protein to lymphocyte ratio

C-reactive protein (CRP) levels were measured via a high-sensitivity immunoturbidimetric assay, while lymphocyte counts were obtained from complete blood count analyses using automated hematology analyzers. CLR was calculated as the ratio of CRP (mg/L) to lymphocyte count (×10^9/L) and categorized into quartiles for comparative analyses.

### 2.4 Covariates

Demographic, socioeconomic, and health-related variables were included as covariates to adjust for potential confounding. These included age, gender, race/ethnicity, education level, marital status, body mass index (BMI), poverty-to-income ratio (PIR), smoking status, alcohol consumption, diabetes, hypertension, and hyperlipidemia.

### 2.5 Statistical analysis

All statistical analyses were performed using SPSS 27.0 and R version 4.4.2. Continuous variables were expressed as mean ± standard deviation (SD) and compared using t-tests, while categorical variables were expressed as frequencies (%) and analyzed using chi-square tests. A two-tailed P-value <0.05 was considered statistically significant. To explore the relationship between CLR and the risk of depression and anxiety, weighted logistic regression models were used, incorporating NHANES sample weights to account for the survey’s complex design. Three models were constructed: Model 1: Unadjusted. Model 2: Adjusted for age and gender. Model 3: Fully adjusted for all covariates. Non-linear associations between CLR and mental health outcomes were evaluated using restricted cubic spline (RCS) regression analyses. These models assessed dose-response relationships and identified potential thresholds for risk. RCS allows for the identification of threshold effects or inflection points where the risk of mental health outcomes changes more sharply. This method is particularly useful when the relationship between independent and dependent variables is not linear, as in the case of CLR and mental health outcomes. Subgroup analyses and interaction tests were conducted to identify effect modifiers, including age, gender, and comorbid conditions. Interaction terms were incorporated into logistic regression models to determine whether the association between CLR and mental health outcomes varied across subgroups.

### 2.6 Ethical considerations

The NHANES protocol was approved by the National Center for Health Statistics Research Ethics Review Board, and all participants provided informed consent. This study utilized publicly available, de-identified data, exempting it from further ethical approval in accordance with the Declaration of Helsinki.

## 3 Results

### 3.1 Baseline characteristics of participants

The study cohort consisted of 11,878 participants for the depression analysis and 11,921 participants for the anxiety analysis, as detailed in **Tables 1**, **2**. The mean age of participants was approximately 51 years, with a slightly higher representation of females (52.4%) than males (47.6%). Significant differences were observed in demographic and clinical characteristics between participants with and without depression or anxiety (P < 0.001 for all variables). For instance, individuals with depression had a younger mean age (49.56 ± 16.89 years) compared to those without (51.57 ± 17.38 years), and a similar trend was observed in the anxiety cohort.

**Table 1:** Baseline characteristics of the study participants.

Characteristics	Overall	Depression		*P*-value
		No	Yes	
n	11878	10715	1163	
Age, years	51.37 ± 17.34	51.57 ± 17.38	49.56 ± 16.89	<0.001
Gender, n (%)				<0.001
Male	5649 (47.6%)	5209 (43.9%)	440 (3.7%)	
Female	6229 (52.4%)	5506 (46.3%)	723 (6.1%)	
Race, n (%)				<0.001
Mexican American	1419 (11.9%)	1286 (10.8%)	133 (1.1%)	
Other Hispanic	1205 (10.2%)	1074 (9.1%)	131 (1.1%)	
Non-Hispanic Black	5481 (46.1%)	4899 (41.2%)	582 (4.9%)	
Non-Hispanic White	2051 (17.3%)	1867 (15.7%)	184 (1.6%)	
Other races	1722 (14.5%)	1589 (13.4%)	133 (1.1%)	
Education, n (%)				<0.001
Less than 9th grade	749 (6.3%)	660 (5.6%)	89 (0.7%)	
9-11th grade	1113 (9.4%)	965 (8.1%)	148 (1.3%)	
High school graduate	2618 (22.0%)	2333 (19.6%)	285 (2.4%)	
Some college or AA degree	3803 (32.0%)	3379 (28.4%)	424 (3.6%)	
College graduate or above	3595 (30.3%)	3378 (28.5%)	217 (1.8%)	
Marital Status, n (%)				<0.001
Married	6254 (52.7%)	5824 (49.1%)	430 (3.6%)	
Widowed	1563 (13.1%)	1358 (11.4%)	205 (1.7%)	
Divorced	1685 (14.2%)	1407 (11.9%)	278 (2.3%)	
Separated	267 (2.2%)	231 (1.9%)	36 (0.3%)	
Never married	1372 (11.6%)	1227 (10.4%)	145 (1.2%)	
Living with partner	737 (6.2%)	668 (5.6%)	69 (0.6%)	
PIR, n (%)				<0.001
<=1	1997 (16.8%)	1662 (14.0%)	335 (2.8%)	
1-3	4966 (41.8%)	4422 (37.2%)	544 (4.6%)	
>3	4915 (41.4%)	4631 (39.0%)	284 (2.4%)	
Smoke, n (%)				<0.001
Yes	5049 (42.5%)	4407 (37.1%)	642 (5.4%)	
No	6829 (57.5%)	6308 (53.1%)	521 (4.4%)	
Alcohol Use, n (%)				<0.001
Yes	9998 (84.2%)	8990 (75.7%)	1008 (8.5%)	
No	1880 (15.8%)	1725 (14.5%)	155 (1.3%)	
Hypertension, n (%)				<0.001
Yes	4373 (36.8%)	3867 (32.5%)	506 (4.3%)	
No	7505 (63.2%)	6848 (57.7%)	657 (5.5%)	
Hyperlipidemia, n (%)				<0.001
Yes	4480 (37.7%)	4003 (33.7%)	477 (4.0%)	
No	7398 (62.3%)	6712 (56.5%)	686 (5.8%)	
Diabetes, n (%)				<0.001
Yes	1754 (14.8%)	1507 (12.7%)	247 (2.1%)	
Borderline	218 (1.8%)	196 (1.6%)	22 (0.2%)	
No	9904 (83.4%)	9010 (75.9%)	894 (7.5%)	
BMI, n (%)				<0.001
Underweight	265 (2.2%)	227 (1.9%)	38 (0.3%)	
Normal weight	3068 (25.9%)	2824 (23.8%)	244 (2.1%)	
Overweight	3841 (32.3%)	3539 (29.8%)	302 (2.5%)	
Obesity	4704 (39.6%)	4125 (34.7%)	579 (4.9%)	

**Table 2:** Baseline characteristics of the study participants.

Characteristics	Overall	Anxious		*P*-value
		No	Yes	
n	11921	6431	5490	
Age, years	51.43 ± 17.36	53.76 ± 17.09	48.70 ± 17.27	
Gender, n (%)				
Male	5675 (47.6%)	3398 (28.5%)	2277 (19.1%)	
Female	6246 (52.4%)	3033 (25.4%)	3213 (27.0%)	
Race, n (%)				
Mexican American	1423 (11.9%)	833 (7.0%)	590 (4.9%)	
Other Hispanic	1210 (10.1%)	673 (5.6%)	537 (4.5%)	
Non-Hispanic Black	5503 (46.2%)	2575 (21.6%)	2928 (24.6%)	
Non-Hispanic White	2059 (17.3%)	1288 (10.8%)	771 (6.5%)	
Other races	1726 (14.5%)	1062 (8.9%)	664 (5.6%)	
Education, n (%)				
Less than 9th grade	757 (6.4%)	484 (4.1%)	273 (2.3%)	
9-11th grade	1125 (9.4%)	656 (5.5%)	469 (3.9%)	
High school graduate	2625 (22.0%)	1473 (12.3%)	1152 (9.7%)	
Some college or AA degree	3817 (32.0%)	1921 (16.1%)	1896 (15.9%)	
College graduate or above	3597 (30.2%)	1897 (15.9%)	1700 (14.3%)	
Marital Status, n (%)				
Married	6275 (52.7%)	3645 (30.6%)	2630 (22.1%)	
Widowed	1579 (13.2%)	838 (7.0%)	741 (6.2%)	
Divorced	1685 (14.1%)	783 (6.6%)	902 (7.5%)	
Separated	267 (2.2%)	120 (1.0%)	147 (1.2%)	
Never married	1377 (11.6%)	686 (5.8%)	691 (5.8%)	
Living with partner	738 (6.2%)	359 (3.0%)	379 (3.2%)	
PIR, n (%)				
<=1	2010 (16.9%)	1014 (8.5%)	996 (8.4%)	
1-3	4987 (41.8%)	2695 (22.6%)	2292 (19.2%)	
>3	4924 (41.3%)	2722 (22.8%)	2202 (18.5%)	
Smoke, n (%)				
Yes	5068 (42.5%)	2517 (21.1%)	2551 (21.4%)	
No	6853 (57.5%)	3914 (32.8%)	2939 (24.7%)	
Alcohol Use, n (%)				
Yes	10017 (84.0%)	5173 (43.4%)	4844 (40.6%)	
No	1904 (16.0%)	1258 (10.6%)	646 (5.4%)	
Hypertension, n (%)				
Yes	4400 (36.9%)	2394 (20.1%)	2006 (16.8%)	
No	7521 (63.1%)	4037 (33.9%)	3484 (29.2%)	
Hyperlipidemia, n (%)				
Yes	4492 (37.7%)	2450 (20.6%)	2042 (17.1%)	
No	7429 (62.3%)	3981 (33.4%)	3448 (28.9%)	
Diabetes, n (%)				
Yes	1768 (14.8%)	1026 (8.6%)	742 (6.2%)	
Borderline	220 (1.9%)	128 (1.1%)	92 (0.8%)	
No	9931 (83.3%)	5276 (44.3%)	4655 (39.0%)	
BMI				
Underweight	266 (2.2%)	108 (0.9%)	158 (1.3%)	
Normal weight	3084 (25.9%)	1657 (13.9%)	1427 (12.0%)	
Overweight	3859 (32.4%)	2225 (18.7%)	1634 (13.7%)	
Obesity	4712 (39.5%)	2441 (20.5%)	2271 (19.0%)	

Educational attainment also varied significantly; participants with depression or anxiety were less likely to have completed higher education. Marital status revealed that never-married individuals and those separated or divorced exhibited higher rates of depression and anxiety. Additionally, comorbid conditions such as hypertension, hyperlipidemia, and diabetes were more prevalent among individuals with mental health symptoms.

### 3.2 Relationship between CLR and depression

The association between the C-reactive protein to lymphocyte ratio (CLR) and depression was assessed through weighted logistic regression models (**Table 3**). In the unadjusted model (Model 1), participants in the highest quartile of CLR (Q4) had significantly higher odds of depression compared to those in the lowest quartile (Q1) (odds ratio [OR]: 1.82; 95% confidence interval [CI]: 1.54–2.16; P < 0.001). After adjusting for age, sex, and race (Model 2), the association remained significant (OR: 1.82; 95% CI: 1.53–2.16; P < 0.001). In the fully adjusted model (Model 3), which accounted for all covariates, the odds ratio was slightly attenuated but remained statistically significant (OR: 1.49; 95% CI: 1.25–1.78; P < 0.001).

**Table 3:** Weighted logistic regression analyses of association between the C-reactive protein to lymphocyte ratio and depression.

CLR	Model 1		Model 2		Model 3	
	OR 95%CI	P value	OR 95%CI	P value	OR 95%CI	P value
Q1	Ref		Ref		Ref	
Q2	0.98 (0.81,1.19)	0.863	1.03 (0.85,1.24)	0.774	0.96 (0.79,1.17)	0.706
Q3	1.29 (1.08,1.54)	0.005	1.32 (1.10,1.58)	0.003	1.16 (0.96,1.39)	0.123
Q4	1.82 (1.54,2.16)	<0.001	1.82 (1.53,2.16)	<0.001	1.49 (1.25,1.78)	<0.001
p for trend	<0.001		<0.001		<0.001	

Model 1: no covariates were adjusted.

Model 2: age, sex, and race were adjusted.

Model 3: age, sex, race, education level, marital status, BMI, PIR, smoking status, alcohol status, diabetes status, hypertension status, hyperlipidemia status was adjusted.

95% CI, 95% confidence interval.

**Figure 2:**
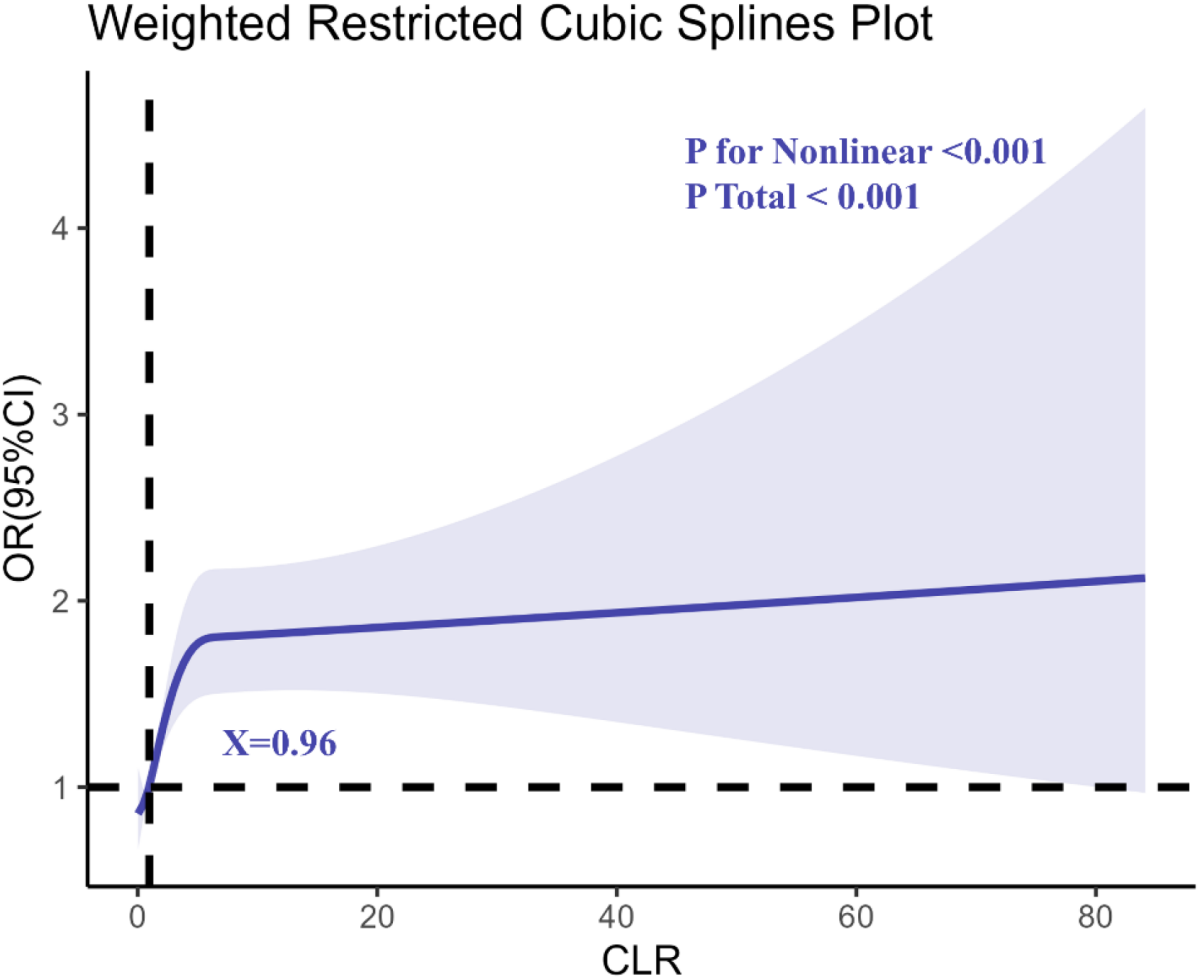
Determination of the association between C-reactive protein to lymphocyte ratio (CLR) and depression by restricted cubic spline (RCS) regression analysis.

Restricted cubic spline (RCS) regression analysis (**Figure 2**) indicated a non-linear dose-response relationship between CLR and depression risk, with a notable inflection point at CLR = 0.96. Beyond this threshold, the risk of depression increased sharply, suggesting a critical level of systemic inflammation associated with elevated mental health risks.

### 3.3 Relationship between CLR and anxiety

Similarly, the weighted logistic regression analysis demonstrated a significant association between CLR and anxiety (**Table 4**). In Model 1, participants in Q4 had a 15% higher likelihood of anxiety compared to Q1 (OR: 1.15; 95% CI: 1.04–1.28; P = 0.006). This association was strengthened after adjusting for age, sex, and race in Model 2 (OR: 1.22; 95% CI: 1.10–1.35; P < 0.001) and persisted in the fully adjusted Model 3 (OR: 1.13; 95% CI: 1.02–1.26; P = 0.023).

**Table 4:** Weighted logistic regression analyses of association between the C-reactive protein to lymphocyte ratio and anxious.

CLR	Model 1		Model 2		Model 3	
	OR 95%CI	P value	OR 95%CI	P value	OR 95%CI	P value
Q1	Ref		Ref		Ref	
Q2	0.89 (0.80,0.98)	0.021	0.97 (0.88,1.08)	0.601	0.93 (0.84,1.03)	0.177
Q3	1.08 (0.98,1.20)	0.127	1.17 (1.05,1.29)	0.004	1.10 (0.99,1.23)	0.069
Q4	1.15 (1.04,1.28)	0.006	1.22 (1.10,1.35)	<0.001	1.13 (1.02,1.26)	0.023
p for trend	<0.001		<0.001		<0.001	

Model 1: no covariates were adjusted.

Model 2: age, sex, and race were adjusted.

Model 3: age, sex, race, education level, marital status, BMI, PIR, smoking status, alcohol status, diabetes status, hypertension status, hyperlipidemia status was adjusted.

95% CI, 95% confidence interval.

**Figure 3:**
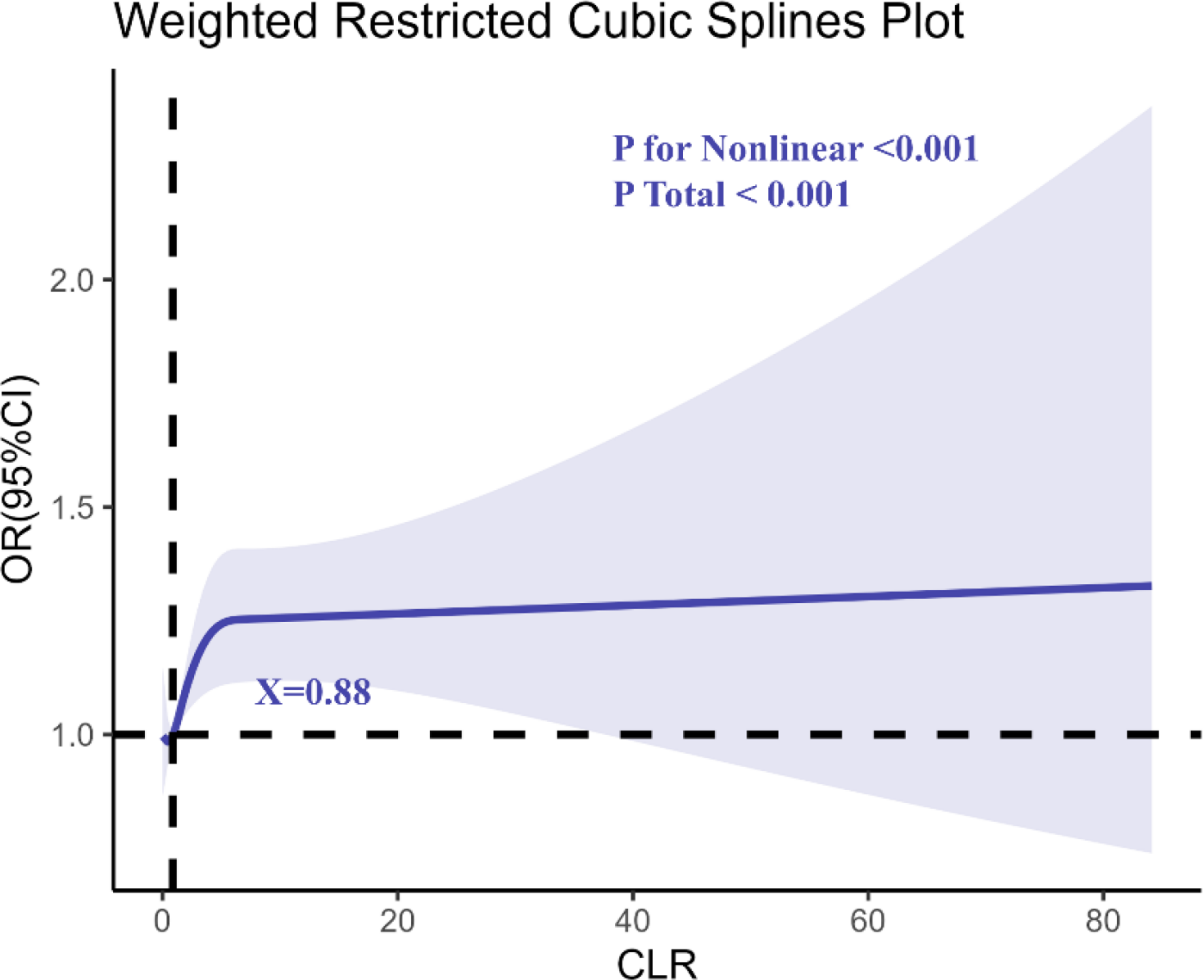
Determination of the association between C-reactive protein to lymphocyte ratio (CLR) and anxiety by restricted cubic spline (RCS) regression analysis.

The RCS regression analysis (**Figure 3**) revealed a non-linear relationship between CLR and anxiety, with an inflection point at CLR = 0.88. This indicates that the risk of anxiety begins to rise steeply beyond this level, highlighting a potential threshold for systemic inflammation contributing to anxiety disorders.

### 3.4 Relationship between CLR and co-occurring depression and anxiety

**Supplementary Table S1** presents the results of the weighted logistic regression analyses examining the association between the C-reactive protein to lymphocyte ratio (CLR) and co-occurring depression and anxiety. The findings from the three models are as follows:

- **Model 1 Q4**: OR 1.67 (95% CI: 1.39, 2.00), p < 0.001
- **Model 2 Q4**: OR 1.67 (95% CI: 1.39, 2.01), p < 0.001
- **Model 3 Q4**: OR 1.31 (95% CI: 1.08, 1.58), p = 0.006

These results indicate a significant association between CLR and the risk of co-occurring depression and anxiety, with the association remaining robust after adjusting for demographic and health-related covariates. Specifically, the fully adjusted model (Model 3) suggests that higher CLR levels are independently associated with an increased risk of co-occurring depression and anxiety, highlighting the potential of CLR as a biomarker for identifying at-risk populations.

**Supplementary Figure S1** illustrates the results of the restricted cubic spline (RCS) regression analysis examining the relationship between the C-reactive protein to lymphocyte ratio (CLR) and co-occurring depression and anxiety. The analysis revealed a critical inflection point at CLR = 0.90, beyond which the relationship between CLR and mental health outcomes shifted from protective to detrimental. Specifically, when CLR was below 0.90, it appeared to serve as a protective factor, whereas CLR values exceeding 0.90 were associated with an increased risk of co-occurring depression and anxiety. This finding emphasizes the non-linear nature of the relationship between systemic inflammation and mental health, suggesting potential clinical implications for identifying at-risk populations based on CLR levels.

### 3.5 Subgroup and interaction analyses

#### 3.5.1 CLR and depression

**Figure 4:**
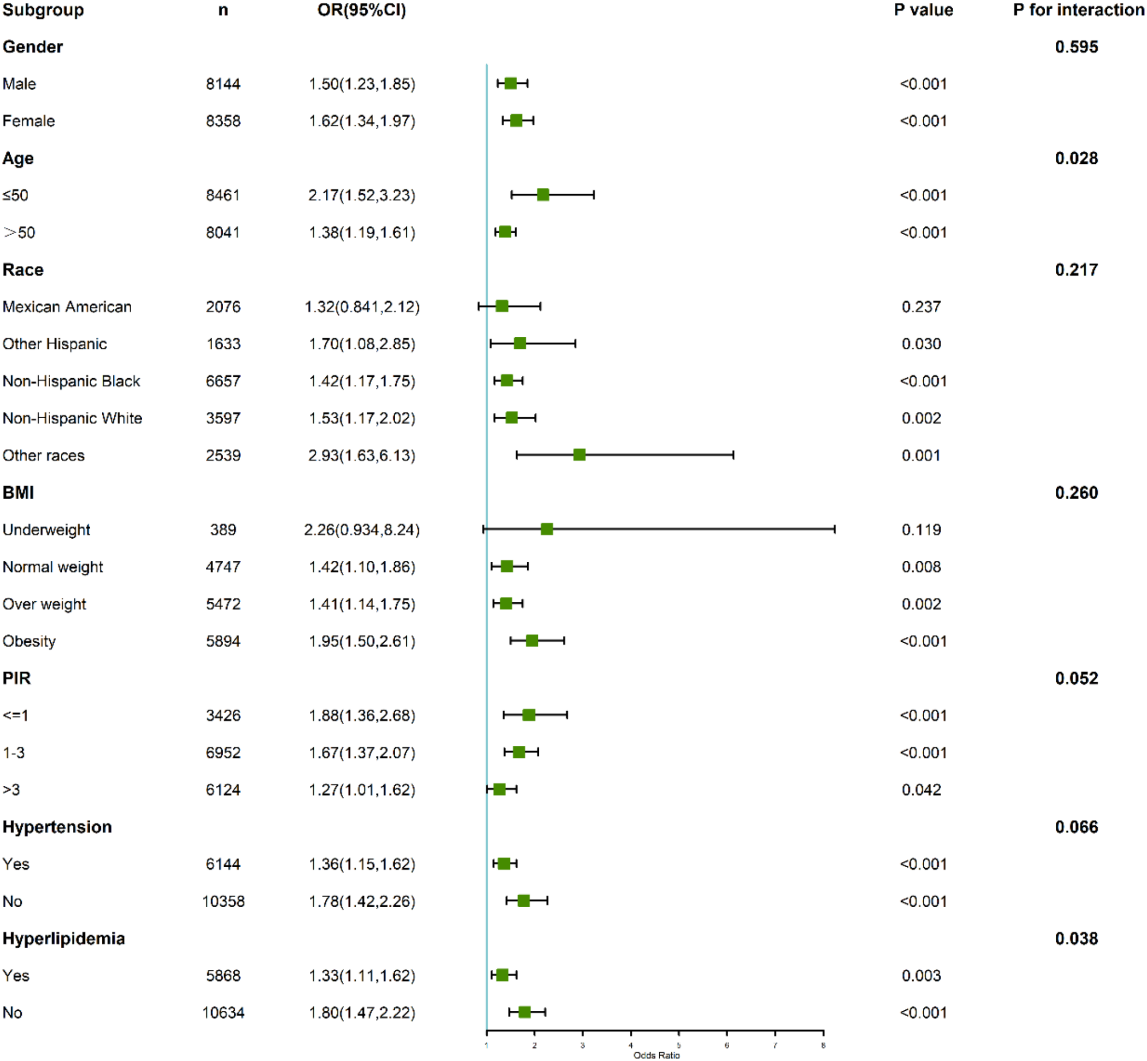
Subgroup and interaction analyses of the association between C-reactive protein to lymphocyte ratio (CLR) and depression.

Subgroup analyses demonstrated significant variations in the relationship between CLR and depression across different demographic and clinical categories (**Figure 4**). Gender-stratified analysis revealed that the association was stronger in females (OR: 1.62; 95% CI: 1.34–1.97; P < 0.001) compared to males (OR: 1.50; 95% CI: 1.23–1.85; P < 0.001), though the interaction test was not statistically significant (P for interaction = 0.595).

Age also modified the relationship, with participants aged ≤50 years showing a heightened association (OR: 2.17; 95% CI: 1.53–3.23; P < 0.001) compared to those older than 50 years (OR: 1.38; 95% CI: 1.19–1.61; P < 0.001). The interaction test confirmed statistical significance (P for interaction = 0.028), indicating that age plays a significant role in modulating the relationship between CLR and depression.

Among individuals with hypertension, the highest CLR quartile was associated with an increased risk of depression (OR: 1.36; 95% CI: 1.15–1.62). Notably, this association was even stronger among those without hypertension (OR: 1.78; 95% CI: 1.42–2.26). The interaction test approached but did not reach statistical significance (P for interaction = 0.066), suggesting a potential modifying effect of hypertension status on the relationship between CLR and depression.

#### 3.5.2 CLR and anxiety

**Figure 5:**
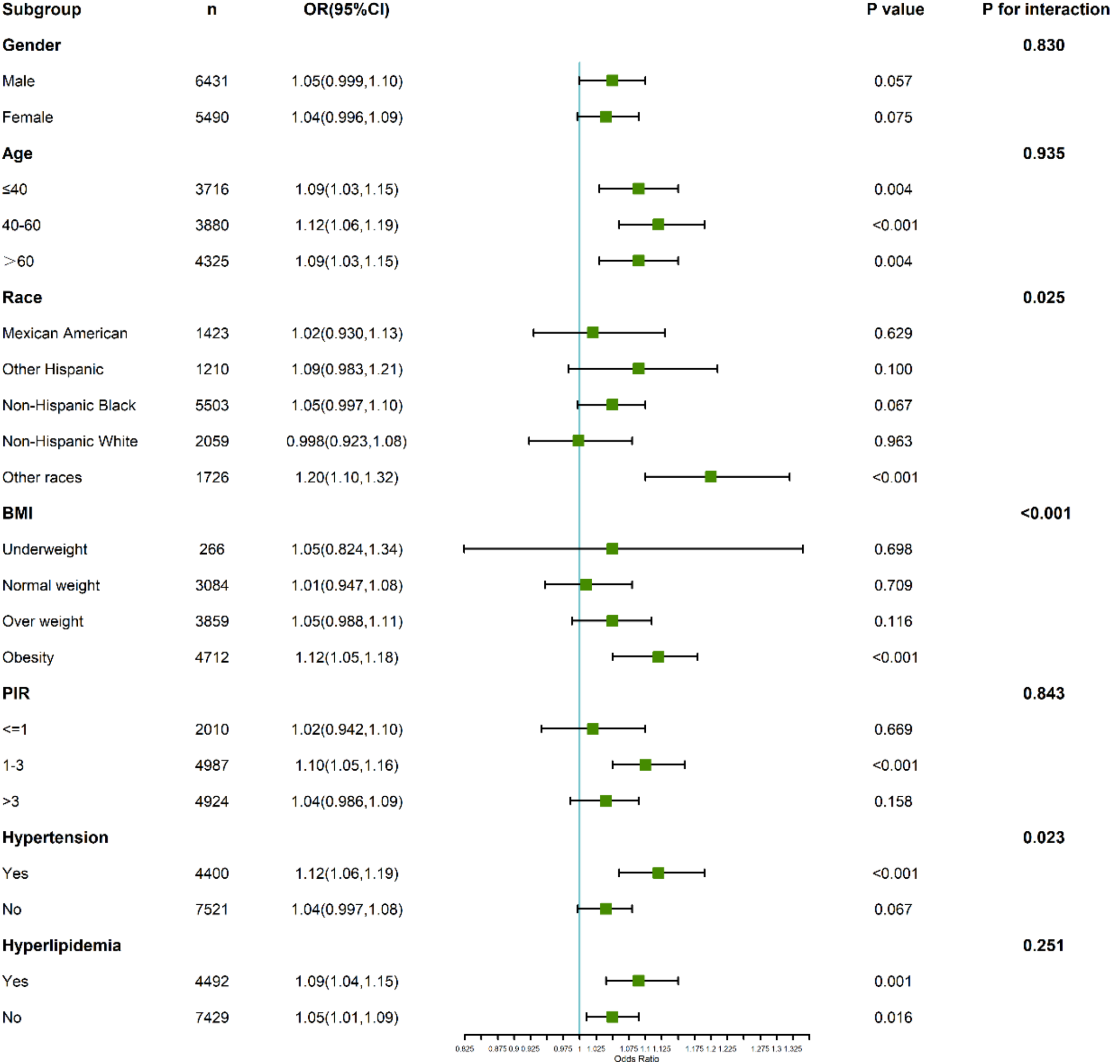
Subgroup and interaction analyses of the association between C-reactive protein to lymphocyte ratio (CLR) and anxiety.

For anxiety, the interaction analysis revealed subtle yet important variations (**Figure 5**). Gender-stratified results showed that males (OR: 1.05; 95% CI: 0.999–1.10; P = 0.057) and females (OR: 1.04; 95% CI: 0.996–1.09; P = 0.075) had similar risk patterns, with no significant interaction (P for interaction = 0.830).

Participants aged ≤40 years demonstrated a stronger association between CLR and anxiety (OR: 1.09; 95% CI: 1.03–1.15; P = 0.004) compared to those above 40 years, where the relationship was attenuated. The interaction test, however, did not reach statistical significance (P for interaction = 0.935).

In individuals with hyperlipidemia, those in the highest CLR quartile exhibited an elevated anxiety risk (OR: 1.22; 95% CI: 1.10–1.35), suggesting a synergistic effect of inflammatory and lipid-related pathways in anxiety pathogenesis. Interaction tests emphasized the need to consider metabolic conditions when interpreting these associations.

#### 3.5.3 CLR and co-occurring depression and anxiety

**Supplementary Figure S2** presents the results of subgroup and interaction analyses exploring the association between the C-reactive protein to lymphocyte ratio (CLR) and co-occurring depression and anxiety. The subgroup analysis revealed that certain groups, including females under the age of 40, Mexican American individuals, obese individuals, and those with hypertension or hyperlipidemia, were more vulnerable to the effects of CLR.

Interaction analysis revealed significant interactions between CLR and body mass index (BMI), as well as poverty-to-income ratio (PIR) (p < 0.05). However, no significant interactions were found between CLR and gender, age, race, hypertension, or hyperlipidemia (p > 0.05). These findings suggest that metabolic factors, such as BMI, and socioeconomic factors, such as PIR, may moderate the relationship between CLR and co-occurring depression and anxiety, highlighting the importance of considering these factors when interpreting the role of inflammation in mental health.

## 4 Discussion

This study provides compelling evidence linking elevated CLR with increased risks of depression and anxiety. The identification of inflection points at CLR = 0.96 for depression and CLR = 0.88 for anxiety emphasizes the critical thresholds of systemic inflammatory burden that disproportionately elevate mental health risks. These findings align with previous research suggesting that systemic inflammation plays a central role in the etiology of mental health disorders (22). Biomarkers like CLR, which capture the balance of pro-inflammatory and immunosuppressive processes, offer novel insights into the complex interplay between physiological and psychological health (23, 24).

Mechanistically, systemic inflammation may disrupt the central nervous system through several pathways (25). Pro-inflammatory cytokines, including interleukin-6 (IL-6) and tumor necrosis factor-alpha (TNF-α), can cross the blood-brain barrier, where they interfere with neurotransmitter metabolism, impair neuroplasticity, and activate microglial cells (26, 27). These processes contribute to structural and functional alterations in brain regions implicated in mood regulation, such as the prefrontal cortex and hippocampus (28, 29). Chronic stress further exacerbates this cycle by activating the hypothalamic-pituitary-adrenal (HPA) axis, leading to sustained glucocorticoid secretion and heightened systemic inflammation (30, 31). The kynurenine pathway is increasingly recognized as a pivotal mechanism linking systemic inflammation to depression. Pro-inflammatory cytokines, such as interleukin-6 (IL-6), have been shown to activate this pathway, leading to the conversion of tryptophan into kynurenine, which subsequently produces neurotoxic metabolites, including quinolinic acid (32). Quinolinic acid, an excitotoxin, can disrupt neurotransmitter systems, particularly by impairing serotonin and glutamate metabolism, which are critical for mood regulation. This metabolic disturbance in the central nervous system is thought to contribute to the development of depressive symptoms (33). Recent studies have provided substantial evidence supporting the role of the kynurenine pathway in the pathophysiology of depression, suggesting that elevated levels of kynurenine and quinolinic acid may serve as biomarkers for inflammation-associated mood disorders (34). Future research could further elucidate how this pathway interacts with other systemic inflammation markers, such as the C-reactive protein to lymphocyte ratio (CLR), to deepen our understanding of the inflammatory underpinnings of depression and potentially guide new therapeutic interventions. These neuroinflammatory pathways have been consistently implicated in both depression and anxiety.

Subgroup analyses in this study revealed important population-specific variations in the CLR-mental health relationship. Younger individuals exhibited a stronger association between CLR and depression, potentially due to a more robust immune response in early adulthood (35). This heightened inflammatory sensitivity may amplify the neurotoxic effects of cytokines, accelerating the onset or progression of mood disorders (36). Similarly, the amplified risk observed in individuals without hypertension suggests that baseline cardiovascular health may modulate the impact of systemic inflammation on mental health (37). These findings highlight the importance of considering demographic and clinical contexts when interpreting the role of inflammatory biomarkers.

For anxiety, the heightened association observed in individuals with hyperlipidemia underscores the synergistic interaction between metabolic and inflammatory dysregulation (38–40). Hyperlipidemia is characterized by the accumulation of pro-inflammatory lipids and cytokines, which may exacerbate systemic inflammation and its downstream effects on the brain (41, 42). This is consistent with prior studies linking metabolic syndrome to increased risks of anxiety disorders, suggesting that targeted interventions addressing metabolic health could mitigate the burden of anxiety (43).

The clinical implications of these findings are substantial. CLR, as an easily measurable and cost-effective biomarker, could serve as a valuable tool for early identification of individuals at risk of depression and anxiety. Integrating CLR measurement into routine health assessments may facilitate targeted interventions, particularly in high-risk groups such as younger adults and individuals with metabolic dysregulation. Anti-inflammatory therapies, including nonsteroidal anti-inflammatory drugs (NSAIDs), and lifestyle modifications, such as increased physical activity and dietary improvements, hold promise in mitigating the inflammatory burden associated with mental health disorders (44–46). In addition to nonsteroidal anti-inflammatory drugs(NSAIDs) and lifestyle modifications, targeting specific cytokines through receptor antagonists, such as IL-6 inhibitors and TNF-α inhibitors, has shown promise in reducing inflammation and improving symptoms of depression and anxiety in some clinical trials. These therapies may offer more targeted approaches for managing inflammation-related mood disorders, although further studies are needed to assess their long-term efficacy and safety in mental health populations (47). Future clinical trials should explore the efficacy of these interventions in reducing CLR levels and improving mental health outcomes.

Despite the strengths of this study, including its large sample size and representative population, certain limitations must be acknowledged. The cross-sectional design precludes causal inferences, and unmeasured confounding factors may have influenced the observed associations (48). Additionally, the use of CLR as a standalone biomarker does not capture the full complexity of systemic inflammation. Combining CLR with other inflammatory markers, such as high-sensitivity CRP or fibrinogen, may enhance its predictive utility (49). While we have adjusted for a range of demographic and health-related variables as covariates, the cross-sectional design of the study prevents us from fully accounting for potential unmeasured confounders. Future longitudinal studies could help further clarify the causal relationship between CLR and mental health outcomes.

## 5 Conclusion

In conclusion, this study highlights the potential of CLR as a biomarker linking systemic inflammation to depression and anxiety. By identifying critical thresholds and subgroup-specific variations, our findings contribute to a deeper understanding of the inflammatory mechanisms underlying mental health disorders. These insights pave the way for targeted prevention and intervention strategies, underscoring the need for integrated approaches to address the systemic nature of mental health challenges.

## Data Availability

Publicly available datasets were analyzed in this study. This data can be found here: https://wwwn.cdc.gov/nchs/nhanes/default.aspx.
