# Supplementary material for: The link between systemic inflammation and mental disorders: a study on CLR, depression, and anxiety in a US cohort
Source: Front Psychiatry. 2025 Aug 18;16:1607982. doi: 10.3389/fpsyt.2025.1607982 (PMC12399671; doi:10.3389/fpsyt.2025.1607982)
Supplement: Supplementary file 1 [file DataSheet1.docx]

Supplementary Table 1. Weighted Logistic Regression Analyses of the Association Between the C-Reactive Protein to Lymphocyte Ratio (CLR) and Co-occurring Depression and Anxiety.

| **CLR** | **Model 1** |  | **Model 2** |  | **Model 3** |  |
| --- | --- | --- | --- | --- | --- | --- |
|  | **OR 95%CI** | **P value** | **OR 95%CI** | **P value** | **OR 95%CI** | **P value** |
| Q1 | Ref |  | Ref |  | Ref |  |
| Q2 | 0.95(0.78,1.16) | 0.617 | 1.01(0.82,1.23) | 0.950 | 0.92(0.74,1.13) | 0.406 |
| Q3 | 1.22(1.01,1.48) | 0.040 | 1.26(1.04,1.53) | 0.021 | 1.07(0.88,1.30) | 0.523 |
| Q4 | 1.67(1.39,2.00) | <0.001 | 1.67(1.39,2.00) | <0.001 | 1.31(1.08,1.58) | 0.006 |
| p for trend | <0.001 | | <0.001 | | <0.001 | |

Model 1: no covariates were adjusted.

Model 2: age, sex, and race were adjusted.

Model 3: age, sex, race, education level, marital status, BMI, PIR, smoking status, alcohol status, diabetes status, hypertension status, hyperlipidemia status was adjusted.

95 % CI, 95 % confidence interval.


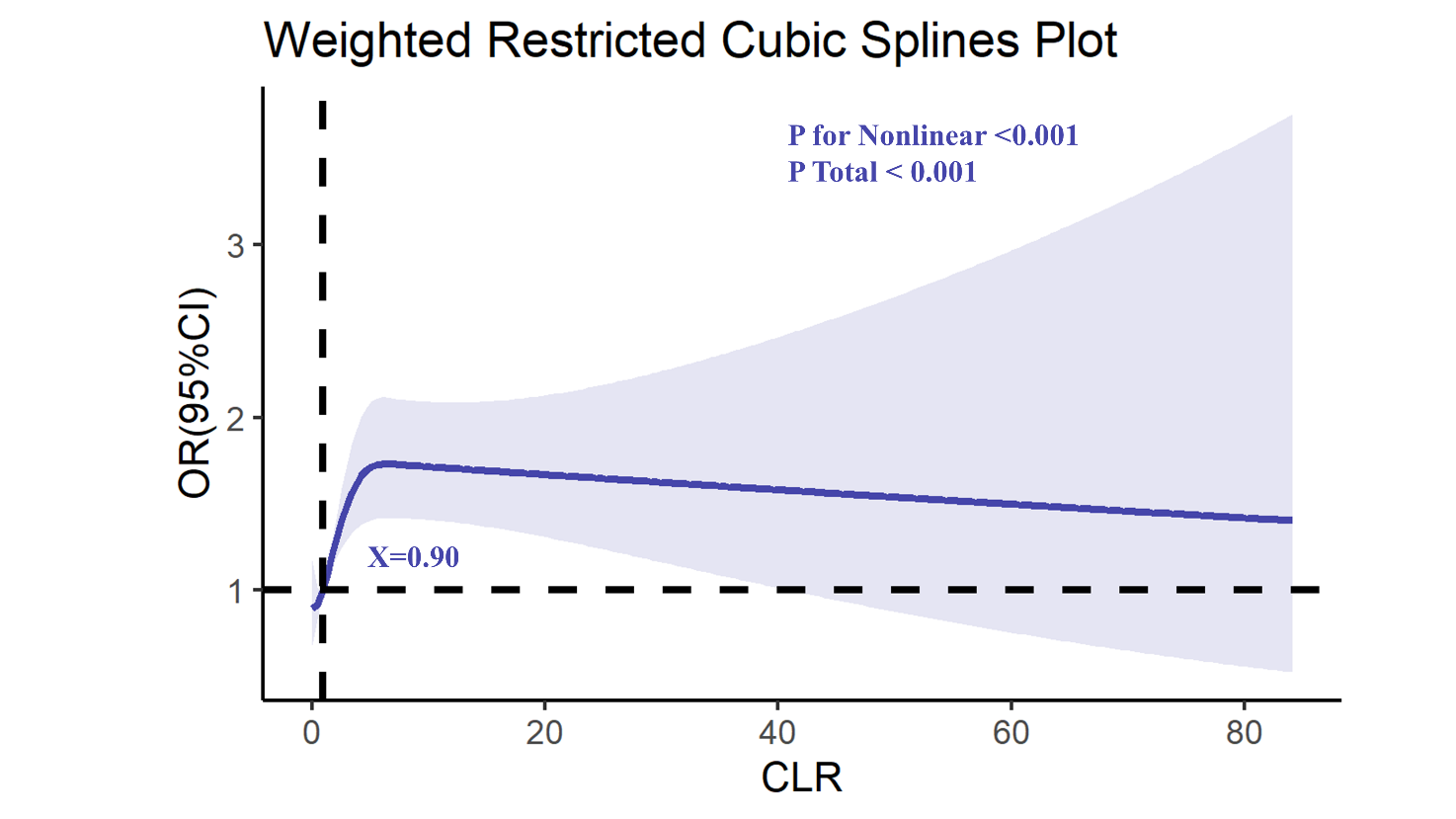
Supplementary Figure 1. Determination of the Association Between C-Reactive Protein to Lymphocyte Ratio (CLR) and Co-occurring Depression and Anxiety by Restricted Cubic Spline (RCS) Regression Analysis


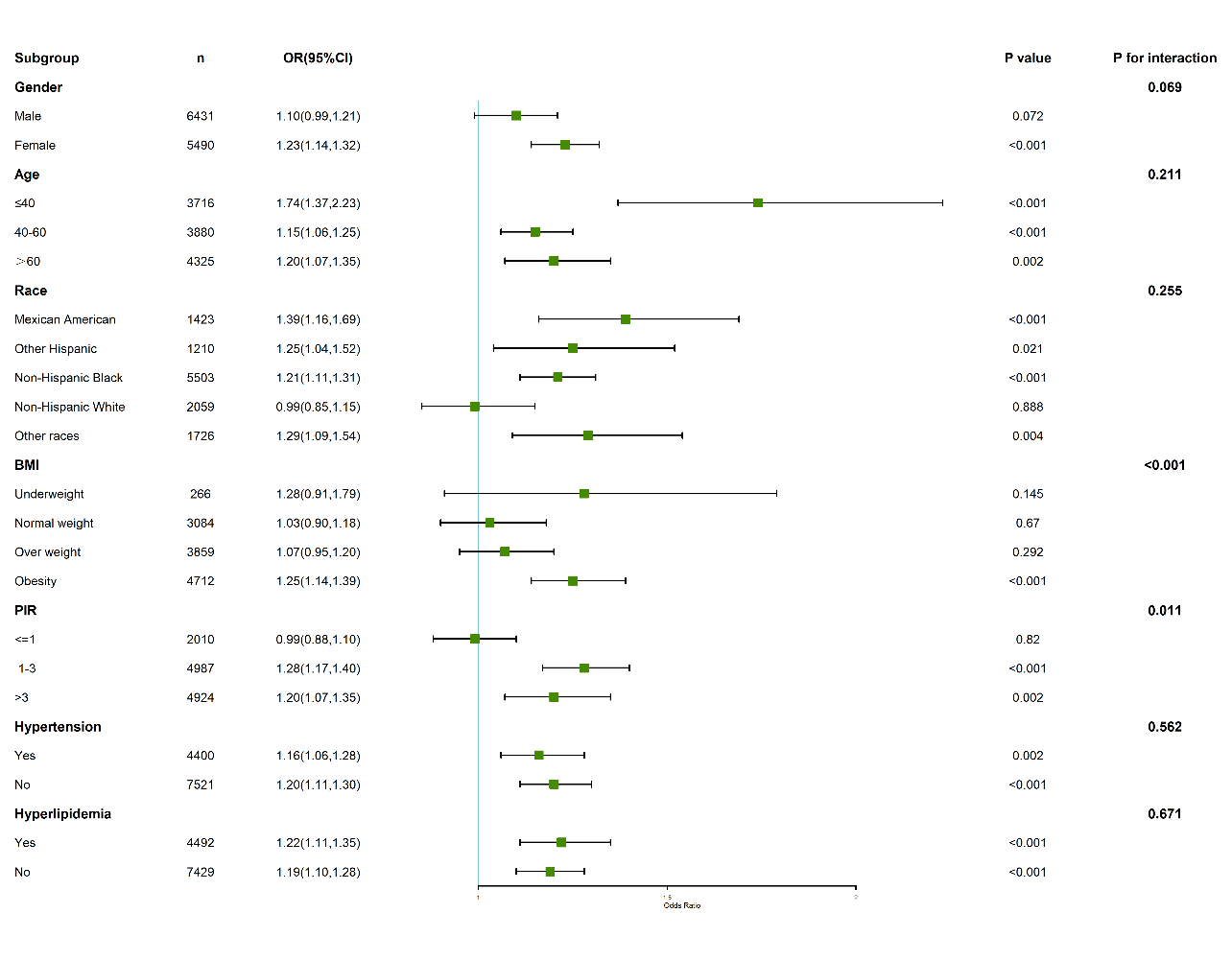


Supplementary Figure 2. Subgroup and Interaction Analyses of the Association Between C-Reactive Protein to Lymphocyte Ratio (CLR) and Co-occurring Depression and Anxiety.
